# Osteopathic Manipulative Treatment for Chronic Neck Stiffness After a Motor Vehicle Collision: A Case Report

**DOI:** 10.7759/cureus.54029

**Published:** 2024-02-11

**Authors:** Jennifer M Jost, Veenah K Stoll, Holly B Waters

**Affiliations:** 1 Department of Basic Sciences, Nova Southeastern University Dr. Kiran C. Patel College of Osteopathic Medicine, Clearwater, USA; 2 Department of Osteopathic Medicine, Nova Southeastern University Dr. Kiran C. Patel College of Osteopathic Medicine, Clearwater, USA

**Keywords:** cervicalgia, motor vehicle accident, motor vehicle collision, chronic neck pain, chronic neck stiffness, osteopathic manipulation, osteopathic manipulative medicine, osteopathy

## Abstract

Neck pain is a multifactorial condition, and one common cause is cervical spine injury related to motor vehicle collision (MVC). Injuries from MVCs range from whiplash to cervical spine fracture and can manifest in various ways including neck stiffness, decreased range of motion, and neurological deficits. One method of management currently underutilized is osteopathic manipulative treatment (OMT), which can be used to treat pain and range of motion deficits resulting from MVCs. While a few studies in the literature have documented a statistically significant benefit of OMT in chronic pain syndromes, there is little data on its effectiveness in treating patients after MVCs.

We present a case of a 25-year-old male who first came to the OMT clinic in January 2021 with complaints of neck pain and stiffness that he attributed to an MVC in February 2020. The collision had led to a loss of consciousness, a concussion, ligamentous injury, and a C5 vertebral fracture. At the OMT clinic, the patient complained of daily headaches associated with “flashes of numbness” throughout his whole body and neck stiffness. The patient was treated initially with a full course of physical therapy, but his symptoms plateaued. He has received OMT about once a month for the past two years. He reported an improved range of motion, no further pain, and decreased neurological symptoms at his most recent visit in October 2023.

There is scarce high-quality research demonstrating the effectiveness of OMT. To the best of our knowledge, this is the first study in the literature to document the use of OMT to treat a patient with a history of cervical fracture with chronic pain and stiffness after an MVC. The closest correlate found during our review of the literature was a case report outlining the successful treatment of post-concussion syndrome after an MVC. Based on the improvement of refractory neck pain and range of motion our patient gained from OMT, further research involving randomized controlled trials needs to be conducted on this topic.

## Introduction

Approximately 869,000 motor vehicle collisions (MVCs)-related cervical spine injuries are treated in hospitals in the United States every year with an estimated 23,500 fractures due to the trauma [1]. MVCs can often lead to cervical spine injury whose severity may range from minor strain or whiplash to serious fracture with spinal cord injury [1]. As the impact load or inertia increases, neck injuries can become increasingly severe or even life-threatening. The combination of forces including post-bending loads and compression forces can cause fractures in both the upper and lower regions of the cervical spine [2]. Whiplash injuries and subsequent fractures can result in neck pain, headache, dizziness, sympathetic nervous disorders, numbness, weakness, cognitive and psychological symptoms, visual disturbances, and other types of symptoms stemming from the head and spine [3]. Neck pain was reported in 100% of patients within 72 hours of a neck injury. Understanding the mechanism behind the initial injury to the neck is vital in clinical decision-making and treatment. Many factors, including the extent and location of the injury, can contribute to the symptoms experienced [3].

Symptoms of a cervical fracture without spinal cord disruption can include significant, localized neck pain and stiffness in a conscious patient. Physical findings can include severe tenderness and decreased range of motion as well as swelling and ecchymosis surrounding the fracture [4]. The management of cervical spine fractures depends on the severity and location of the fracture. Initial management is important for the long-term prognosis of patients and begins with early and effective pain control, but the main focus is on stabilization of the spine with bracing or surgery [3,4]. For the lower cervical spine, ligamentous and bony structures play an equal role in the stability of the spine. Stable minor fractures can be treated nonoperatively with cervical bracing [3,5]. This necessary prolonged stabilization can result in atrophy of the musculature and ligamentous structures, leading to a decreased range of motion and stiffness.

Recovery usually occurs within a few weeks to months, but a subset of patients continue to suffer years after the initial injury. The Quebec Task Force issued guidelines to standardize the treatment of whiplash-associated disorders. After one week, return to usual activities is encouraged with consideration for manual or physical therapy. Management by a multidisciplinary pain team or rehabilitation provider is recommended if the condition fails to resolve after 12 weeks [3].

## Case presentation

A 25-year-old male with a past medical history of Tourette syndrome, migraines, and dysautonomia presented to the osteopathic manipulative treatment (OMT) clinic complaining of neck pain and stiffness. These symptoms had reportedly begun following a severe MVC in February 2020. He stated that he was driving in the rain when he hit a disabled tractor-trailer on a corner. His vehicle spun out and had been hit by multiple other cars. While no medical records were available, the patient stated that he had been diagnosed with a concussion and a non-displaced C5 vertebral body fracture during the initial emergency department visit with subsequent post-concussion syndrome, tiredness, emotional lability, and depressed mood, all of which had resolved within a year of the accident and before his OMT treatments.

The patient completed a full course of physical therapy, but progress plateaued with continued neck stiffness and limited range of motion. He was then referred for OMT. He also followed up with neurology and behavioral health for his comorbid neurologic and psychiatric conditions.

Upon presenting for OMT in January 2021, he complained of moderate neck stiffness associated with headache and “flashes of numbness” throughout his entire body. The neck stiffness was present all day most days and was associated with pain that he rated a 2/10 and subjective decreased range of motion on right neck rotation. He denied any radiation down the arm, tingling of his extremities, or weakness in the extremities. On the physical exam, the pain was found to exacerbate with spinal extension. A neurological exam revealed no abnormalities. A full osteopathic exam from his first visit on 1/8/2021 compared to his most recent visit on 10/9/2023 is detailed in Table 1.

**Table 1:** Summary of the full osteopathic structural exam on the first encounter and during the most recent visit organized by body region

Body area	Diagnoses on 1/8/2021	Diagnoses on 10/9/2023
Head and cranium	Right cranial sidebending rotation with some degree of vault compression	Left cranial sidebending rotation, left lateral strain
Occipitoatlantal joint	Extended sidebent right rotated left	Extended sidebent right rotated left
Cervical spine	C2 flexed sidebent left rotated left, C3-5 flexed sidebent right rotated right	C2-4 extended sidebent left rotated left, C5 with interosseous strain, bilateral scalene hypertonicity
Thoracic spine	T1-2 extended sidebent left rotated left, T4-6 flexed sidebent right rotated right with decreased compliance	T1-5 neutral sidebent left rotated right, T6-12 neutral sidebent right rotated left
Lumbar spine	Flattened lordosis, decreased compliance at L1-3	L1-4 neutral sidebent left rotated right, bilateral quadratus lumborum hypertonicity
Sacrum	Left sacroiliac joint restriction	Base posterior
Pelvis	Left superior innominate shear	Left anterior rotation, inflare, right > left pelvic mesenteric drag
Ribs	Right rib 1 inhaled, left rib 12 inhaled	Right rib 1 inhaled, right rib 12 inhaled
Upper extremity	Right deltoid hypertonicity, right > left trapezius hypertonicity	Bilateral shoulder retraction
Lower extremity	Left hip externally rotated	Right hip externally rotated, left iliac counterstrain tenderpoint
Abdomen	Left > right hemidiaphragm inhaled severely	Right > left hemidiaphragm inhaled

The patient consented to undergo OMT for his stiffness and decreased range of motion. The techniques utilized included osteopathy in the cranial field, muscle energy technique (MET), myofascial release (MFR), balanced ligamentous tension (BLT), articulatory, and counterstrain (CS) techniques. These various techniques were applied throughout the body including the cervical spine, thoracic spine, and lumbar spine. In addition to treating these areas, the sacrum, pelvis, and extremities were treated. Gentle techniques such as CS and indirect MFR were preferentially used in this patient as it was felt that more aggressive techniques such as high-velocity low-amplitude maneuvers would have limited benefit given his history of chronic pain and previous fracture. At-home stretches including a scalene muscle stretch bilaterally for two to three minutes twice daily were also recommended to the patient.

The dates on which the patient received treatment were as follows: 1/8/2021, 1/15/2021, 1/29/2021, 3/30/2021, 5/5/2021, 6/14/2021, 6/28/2021, 7/26/2021, 9/1/2021, 9/15/2021,10/18/2021, 11/24/2021, 1/7/2022, 2/14/2022, 3/28/2022, 5/2/2022, 6/1/2022, 7/6/2022, 8/3/2022, 9/9/2022, 10/3/2022, 10/28/2022, 11/30/2022, 1/11/2023, 3/6/2023, 7/31/2023, 9/11/2023, and 10/9/2023. During each visit, MET, cranial, MFR, BLT, and articulatory techniques were used to treat the dysfunctions, as summarized in Table 1. After the first session, the physician noted improvement in the range of motion specifically with right rotation of the cervical spine, and the patient also noted improvement in symptoms. At his second visit one week later, the patient reported significant improvement in pain, stiffness, and range of motion. By the sixth appointment in June of 2021, he reported complete resolution of pain, numbness, and tingling with improved stiffness. Monthly treatment was recommended, and he reported good compliance with his home stretches. Since his most recent visit in October 2023, he reported that his symptoms are stable at an overall improved baseline with slight exacerbation towards the end of the four- to six-week interval between treatments.

## Discussion

Neck pain is a multifactorial issue that often leads to a significant economic burden due to increased healthcare spending as well as work losses. MVCs have been a common cause of this complaint since the invention of the automobile. More than 800,000 MVCs involving neck injuries are reported in the United States annually [2]. Many patients will experience similar symptoms of neck pain, stiffness, numbness, tingling, and headaches as those experienced by the patient in this case.

While this condition is commonly diagnosed in the acute setting following the initial trauma, patients may present weeks to years after the initial injury as the body’s natural compensation patterns evolve. A detailed view of the soft tissue with an MRI is clinically useful to determine the presence and location of any ligamentous injury or swelling [6]. MRI is the imaging modality of choice for patients who complain of neurological symptoms [6], but a CT scan remains the gold standard imaging for diagnosing fractures in an acute setting [5].

Chiropractic care, physical therapy, nonsteroidal anti-inflammatory medication, and muscle relaxers are commonly used as treatment for chronic neck pain and stiffness after an MVC. While there is no formal literature describing OMT in the treatment of neck pain following a cervical fracture after an MVC, some case reports have described the successful use of OMT for the treatment of whiplash-associated disorders and post-concussion syndrome [7-9]. It has also been used in the treatment of chronic neck pain with positive outcomes [10]. To the best of our knowledge, this is the first report in the literature to document the use of OMT in a patient with a cervical fracture, chronic neck pain, and stiffness after an MVC. While the findings in the current case have limited value, this case provides a pertinent example of a gentle treatment plan incorporating OMT.

This report has a few limitations. No standardized assessment tool was used to quantify the range of motion or pain improvement, and hence only subjective patient experience is described. Another limitation involves the lack of records and imaging from the initial hospital visit following the MVC. The patient stated that initial imaging had been performed during his emergency department visit immediately after the MVC, but records were not available for review. Given the absence of further injury or any worsening of symptoms, additional imaging was not performed.

## Conclusions

A comprehensive approach to patients with a prior cervical fracture, including OMT, can help provide symptom relief and improve outcomes. OMT is a cost-effective treatment option that can be used to target stiffness, and pain, as well as the musculature, ligamentous structures, and supporting lymphatic systems. In our patient, regular treatment with OMT combined with the use of home stretches of the scalenes bilaterally led to improvement in pain and range of motion. We recommend further research involving randomized controlled trials to gain deeper insights into the effectiveness of OMT by using objective, validated measures.
